# Supplementary material for: MetaRibo-Seq measures translation in microbiomes
Source: Nat Commun. 2020 Jun 29;11:3268. doi: 10.1038/s41467-020-17081-z (PMC7324362; doi:10.1038/s41467-020-17081-z)
Supplement: Supplementary file 10 — Supplementary Data 7 [file 41467_2020_17081_MOESM10_ESM.zip › File2/Confidence_High_Taxonomy/225632_out.krona.html]

Javascript must be enabled to view this page.

members
magnitude
magnitudeUnassigned
count
unassigned
taxon
rank

225632\_out

7

7
2
superkingdom

phylum
976
7

class
200643
7

7
171549
order

815
family
7

7
genus
816

SRS014235\_contig\_number\_20667
1

2292941
species
3

SRS016989\_contig\_number\_6755SRS052027\_contig\_number\_11517SRS076756\_contig\_number\_11767

1
species
626929

SRS1041164\_contig\_number\_418

204516
species
1

SRS893172\_contig\_number\_13687


SRS020233\_contig\_number\_contig-100\_6744.132840
1
1841855
species
